# Bunny Burrito Restraint for Computed Tomography in Conscious Rabbits: An Effective Alternative

**DOI:** 10.1111/vru.70230

**Published:** 2026-08-26

**Authors:** Hung‐Ting Liu, Jonathan Olijnyk, Jessica Ingman, Ellen Sundberg, Laetitia Volait‐Rosset, Alexis Gombert

**Affiliations:** ^1^ Evidensia Uppsala Veterinary Clinic Uppsala Sweden; ^2^ Diagnostic Imaging Department Department of Clinical Sciences Swedish University of Agricultural Sciences Uppsala Sweden; ^3^ Evidensia Roslagstull Veterinary Clinic Stockholm Sweden

**Keywords:** computed tomography (CT), nonchemical restraint, *Oryctolagus cuniculus*, rabbit handling, veterinary imaging techniques

## Abstract

Computed tomography (CT) is a critical diagnostic tool in rabbits. Traditional CT imaging often requires sedation or general anesthesia, posing inherent risks such as mortality and gastrointestinal complications. An effective physical restraint method could enhance patient safety, reduce costs, and improve accessibility to CT imaging. This study evaluates the effectiveness of the well‐known bunny burrito restraint technique as an alternative to the plexiglass chamber method for conducting CT examinations in conscious rabbits. A retrospective multicenter observational study was conducted on 93 client‐owned rabbits undergoing CT between March 2021 and December 2023. Cases were classified into three restraint groups: bunny burrito (*n* = 36), plexiglass chamber (*n* = 47), and sedation (*n* = 10). CT image quality was assessed on the basis of motion artifacts, positioning, and stability across different acquisitions. Statistical analyses, including logistic regression, were performed to compare the two physical restraint methods. The bunny burrito technique had significantly lower motion artifacts in pre‐contrast acquisition (*p* = 0.02), better positioning in the dental disease subgroup (*p* = 0.03), and better stability in early post‐contrast period (*p* = 0.04, wide confidence interval) compared to the plexiglass chamber group. Image quality was similar between bunny burrito and sedation in descriptive results. No complications were reported during or after CT examinations. In conclusion, the bunny burrito method is an effective and safe restraint method for CT imaging in conscious rabbits that shows advantages over the plexiglass chamber in specific imaging contexts. It minimizes sedation‐related risks with simplified equipment. This method represents a valuable advancement in veterinary diagnostic imaging.

## 1 Introduction

Computed tomography (CT) is an important diagnostic tool for rabbits. The high spatial resolution and multiplane capabilities of CT imaging afford meticulous examination, offering a level of precision essential for effective diagnosis in these patients. Investigations have demonstrated the superior diagnostic efficacy of CT over conventional radiography, particularly in the assessment of dental, auricular, and intranasal pathologies [1, 2, 3, 4]. Moreover, CT holds promise in enhancing the diagnostic capability for abdominal and respiratory pathologies in rabbits, given the constraints associated with commonly utilized modalities such as ultrasound and radiography in these anatomical regions [5, 6].

General anesthesia or sedation was until recently deemed necessary to chemically restrain animals for the acquisition of satisfactory diagnostic CT images devoid of motion artifact [7]. However, general anesthesia in rabbits entails inherent risks. Reported overall mortality rates during anesthesia in healthy rabbits exceed those observed in dogs and cats, with systemic illnesses further exacerbating mortality [8]. Peri‐ and postanesthetic periods commonly present gastrointestinal complications, such as gastrointestinal stasis syndrome [9]. Considerable time and resources are required for anesthesia preparation and post‐procedural recovery. Therefore, implementing a secure physical restraint method is paramount to minimize sedative use in rabbits during CT examinations. This approach not only enhances patient safety and reduces complications but also broadens access to the diagnostic accuracy of CT examinations by reducing costs and examination duration.

In the context of feline and small canine patients, a commercially available plexiglass chamber has demonstrated efficacy in restraining and positioning patients without sedation or with minimal sedation [10, 11]. Similarly, utilization of a plexiglass chamber has proven successful in facilitating CT examinations in rabbits presenting with diverse clinical concerns [12, 13]. However, the availability of these commercial plexiglass devices remains limited, and recurrent motion artifacts can limit acquisition quality [11]. These different limitations motivated us to explore another solution.

Wrapping rabbits in a towel, also referred to as the bunny burrito, is a well‐known technique for safe handling during clinical examinations [14]. It takes advantage of the natural tendency of rabbits to remain still, when snugly wrapped. In this study, we evaluated the use of the bunny burrito method as a restraint solution to conduct CT examinations without necessitating sedation in rabbit patients. Our study aimed to assess the resulting CT image quality associated with this method and compared it to plexiglass chamber. We hypothesized that the bunny burrito would be an effective method allowing CT studies in conscious rabbits and would achieve better CT image quality to plexiglass chamber restraint, while maintaining diagnostic outcomes comparable to chemical restraint.

## 2 Methods

### 2.1 Subject Selection and Description

This was a retrospective observational study. All client‐owned rabbits that underwent a CT examination for diagnostic purposes in the University Animal Hospital of Swedish University of Agricultural Sciences (Institution 1), Evidensia Uppsala Veterinärklinik (Institution 2), and Evidensia Södra Djursjukhuset Kungens Kurva (Institution 3) between March 1, 2021 and December 31, 2023 were reviewed in the digital journal systems. The inclusion criteria were limited to CT studies encompassing the skull. Acquisitions were excluded if restraint method could not be identified. If the same individual was scanned on two occasions, only the earliest set of acquisitions was included.

### 2.2 Data Recording and Analysis

#### 2.2.1 Medical Record Review

The rabbits’ medical histories were reviewed by a specialized intern in diagnostic imaging (H‐T.L.) and an imaging technician (E.S.), aware of the hypothesis. Age, body weight, sex, breed, reason of study, diagnosis in the original imaging reports, restraint type, and any adverse event during or 24 h after the CT study were documented. Use of data was approved by each hospital. Institutional animal care and use committee approval was not needed for medical records review.

#### 2.2.2 Restraint Methods

##### 2.2.2.1 Bunny Burrito

**FIGURE 1:**
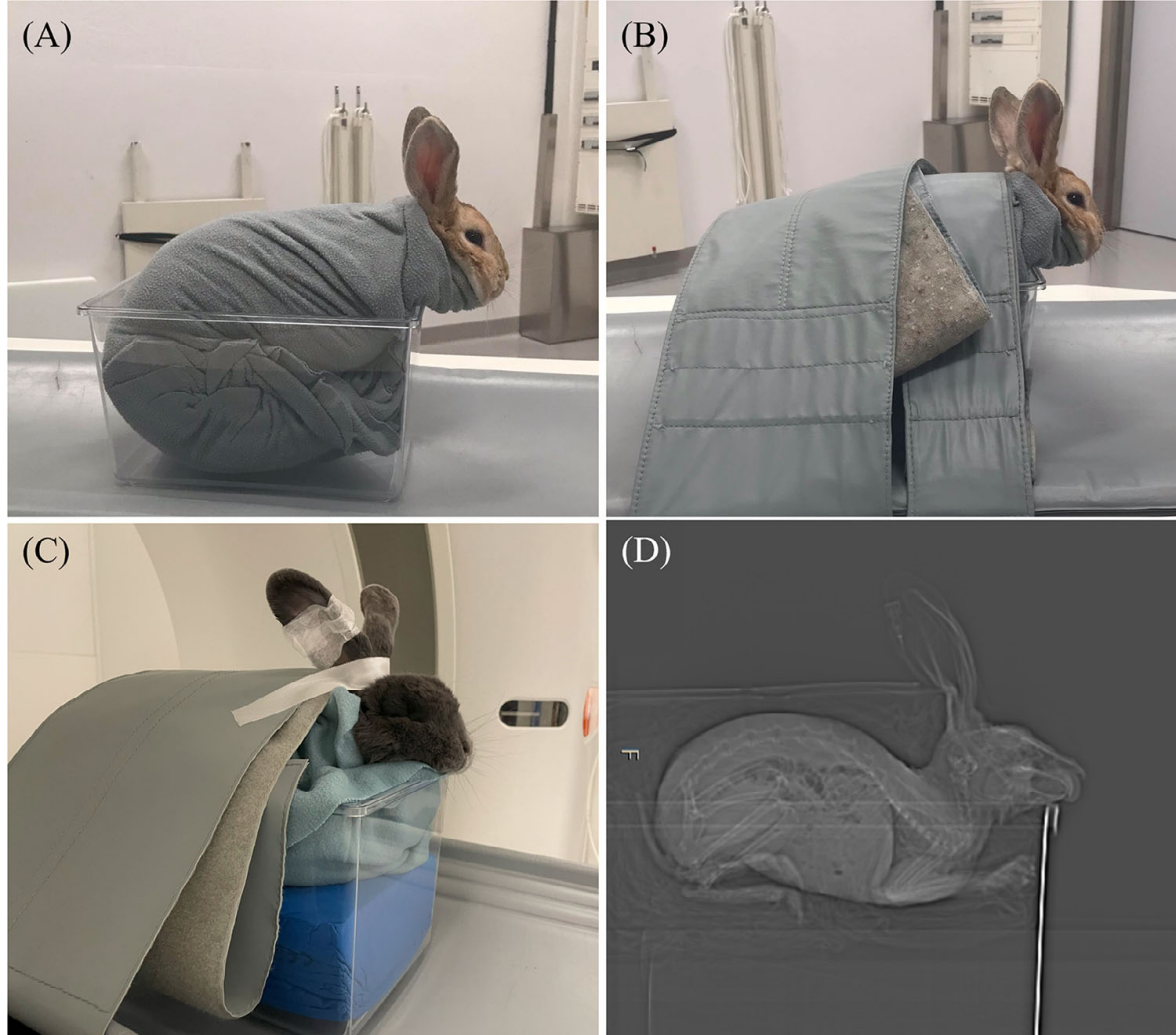
Example of rabbits contained by the bunny burrito restraint. (A) Rabbits were first wrapped with a soft fabric and positioned in an open plastic box. (B) The straps of CT table were placed over rabbits behind the head. (C) For smaller rabbits, additional foam can be placed at the bottom of the box. On some patients, the ears were taped upright. (D) A sagittal topogram scan shows a rabbit maintaining its natural resting position in the burrito method setting.

The rabbits were wrapped in a soft fabric before being placed into a suitable open plastic box in sternal resting position with their mandible resting on one edge of the box. The boxes were standard pet store transport/observation containers for small pets (26 cm length, 16 cm width, and 17 cm height). Additional soft fabric or foam blocks were added to the bottom of the box if the rabbits were too short to rest their mandible on the edge. The hook‐and‐loop straps of the CT table were then placed above the box and the rabbits, exposing the head and ears to secure the box and prevent them from jumping upward (Figure 1). The ears of rabbit with an auricular catheter were positioned in front of the straps. Where needed, the ears were loosely taped to the straps to prevent cranial folding and facilitate contrast passage. CT was performed in a quiet room, with efforts made to dim the lights and handle the rabbits gently to minimize anxiety. This bunny burrito technique was developed by an imaging technician (E.S.) from Institution 1, where it had been the sole restraint method since December 2022. The technique was carried to the other institutions by personnel migration. All involved technicians have more than 5 years of experience and are trained in burrito wrapping.

##### 2.2.2.2 Custom‐Made Cylindrical Plexiglass Chamber

**FIGURE 2:**
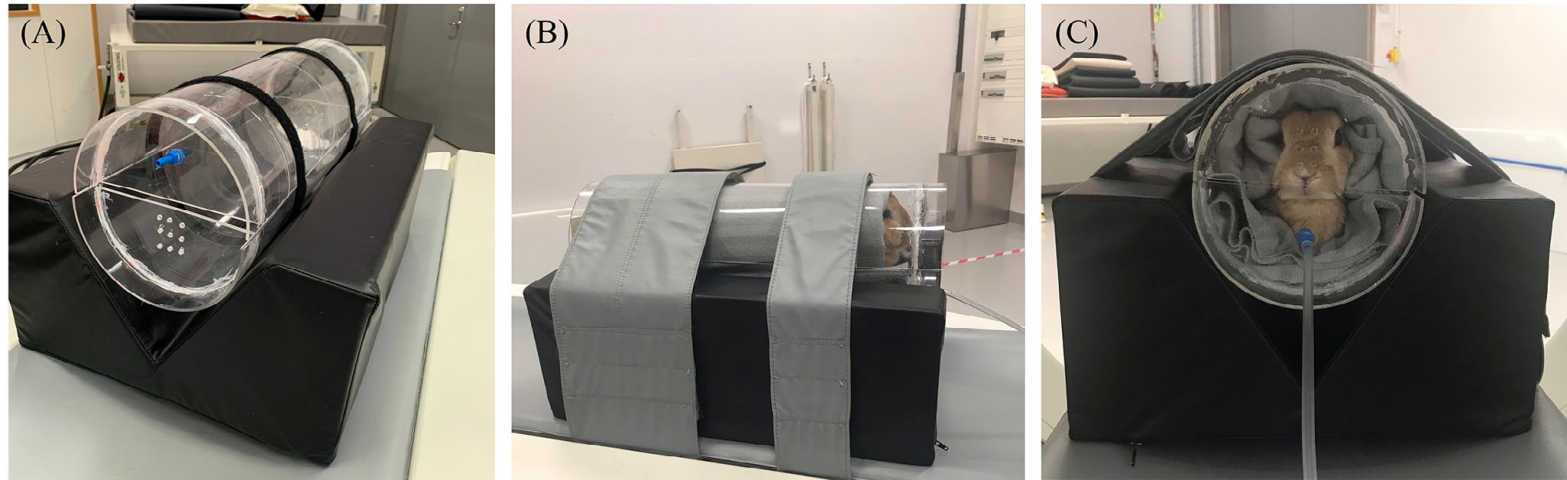
Example of a rabbit contained by plexiglass chamber method. (A) The plexiglass chamber was specifically fabricated for Institution 1 with two hook‐and‐loop straps holding the lid. The chamber is placed on top of a positioning cushion. (B) Lateral view of the chamber with a rabbit inside, surrounded by soft fabric. The straps of CT table secure the chamber. (C) Frontal view of the chamber with a rabbit inside and oxygen tube at front.

Rabbits were positioned in a closed cylindrical plexiglass chamber (48 cm length and 20 cm diameter) that had been specifically fabricated for Institution 1. The chamber has small round apertures for breathing, and ports for catheter access. It was positioned on a foam wedge for stability. Rabbits were placed in sternal resting position in midline of the restraint device, bounded by soft fabric except at the front of the tube for oxygen access (Figure 2). The same measures for reducing anxiety as in the previous method were implemented.

##### 2.2.2.3 Sedation

Sedation protocols were selected on the basis of clinician preferences. The list of the used agents is in Supporting Information S1. Flow by oxygen was provided as necessary. When possible, the sedation was reversed at the end of the procedure. The rabbits were positioned in sternal recumbency along and aligned with the long axis of the CT scan table.

#### 2.2.3 Image Acquisition

Images were acquired with three different multidetector CT scanners: a 32‐slice unit (Somatom go.All, Siemens Healthcare, Erlangen, Germany), a 64‐slice unit (Definition, Siemens Healthcare, Erlangen, Germany), and a 128‐slice unit (Ingenuity Core 128, Philips Healthcare, Best, Netherlands). CT settings were as follows: pitch of 0.35–1.2 in Institution 1 (following scan protocol choice), 0.55 in Institution 2, and 0.398 in Institution 3, tube potential of 120 kVp, reference tube current of 55–139 mA, slice thickness of 0.6–1 mm, and matrix 512 × 512. If intravenous contrast was indicated, a bolus of 600 mg iodine/kg of nonionic iodinated contrast medium (Omnipaque 300 or 350 mgI/mL, GE healthcare, Oslo, Norway) was manually injected through an intravenous catheter in marginal ear vein, followed by 2 mL of saline solution flush. Post‐contrast images were acquired, immediately and within 2 min after contrast administration in all institutions. Acquisitions began only after all personnel left the CT room, with any repositioning or procedures only performed between acquisitions. Additional acquisitions were acquired if images were nondiagnostic or dacryocystography was needed.

#### 2.2.4 Image Assessment

**FIGURE 3:**
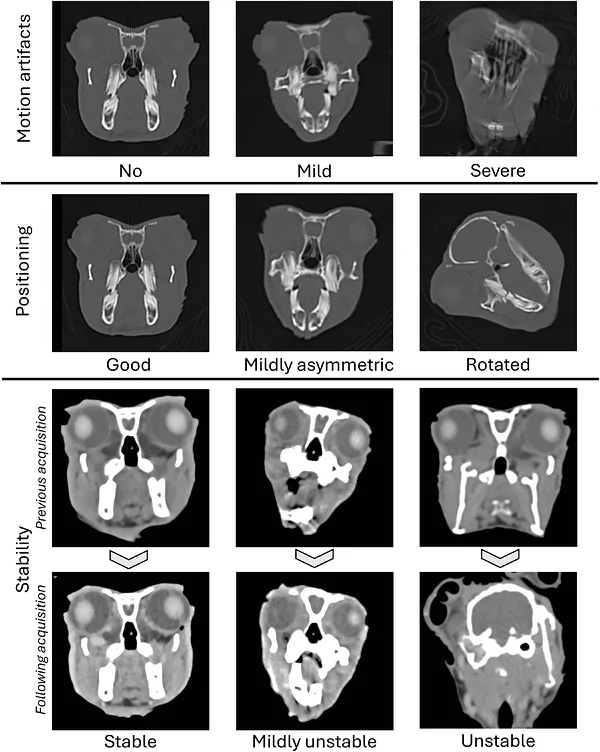
Grading scheme of motion artifacts, positioning, and stability. Images were graded in bone window for motion artifacts and positioning, and in soft tissue window for stability as indicated above. All images were graded in unadjusted transverse plan.

Images were reconstructed with low‐ and high‐frequency algorithms, then anonymized, and randomized ensuring that pre‐ and post‐contrast images were paired together. All images were assessed in consensus in DICOM format using standard medical imaging viewing software (Horos, Horos Project) simultaneously in one session by two ECVDI diplomates (A.G. and J.I.) and one imaging intern with 1 year of CT experience (H‐T.L.). Subjective image quality assessment was performed only on the head section of the CT images for better repeatability. Each sequence was first evaluated according to the degree of motion artifacts in bone window for maximized sensitivity: it was graded as “no” if no motion artifact was identified, “mild” when motion artifacts were present but did not affect image interpretation, and “severe” if motion artifacts hindered interpretation. The position of the head relative to the plane of the gantry during the first acquisition (hereafter referred to as the pre‐contrast acquisition) was also assessed in bone window: it was considered “good” positioning if left/right symmetry was evident, “mildly asymmetric” if a slight left/right discrepancy was noted but eyes or tympanic bullae could still be identified in the same transverse plane, and “rotated” if clear obliquity was observed. For cases where subsequent acquisitions (hereafter referred to as post‐contrast acquisitions) were performed, the stability of the rabbits’ head position between acquisitions was evaluated in soft tissue window, as most post‐contrast acquisitions were available only in soft tissue reconstructions as soft tissue enhancement was the main purpose for contrast studies: It was classified as “stable” if no changes were noted, “mildly unstable” if only slight positional change was detected, which did not hinder comparison between acquisitions, and “unstable” if significant positional change was identified (Figure 3). A predesigned Microsoft Excel spreadsheet was used to record the gradings. Study duration was defined by the time from the beginning of the first scan acquisition to the end of the last scan acquisition, retrieved from the DICOM metadata, not including patient setup time.

### 2.3 Statistical Analysis

The statistical analysis was conducted by a veterinarian with postgraduate education in statistics (J.O.) using R version 4.3.3 (Angel Food Cake). Data were formatted and cleaned, with removal of repeat studies and those with incomplete imaging data. Sedated rabbits were excluded from the statistical analysis and received no formal hypothesis testing.

Continuous variables were summarized using medians and interquartile ranges and assessed for normality using visual inspection of Q‐Q plots and the Shapiro–Wilk test. Categorical variables were summarized using frequencies and percentages. Distributions of the grades of total motion artifacts, positioning, and total stability were plotted for their available acquisitions. Fisher's exact tests were used to assess associations between restraint method and categorical demographic variables. Agreement between presenting complaints and final diagnoses for auricular and dental disease was evaluated using exact McNemar's tests.

Primary outcomes (motion artifacts, positioning, and stability) were analyzed using logistic regression models. Mixed models were used to account for repeated measures within individuals (rabbit ID as a random intercept). Grades of movement, positioning, and stability were altered into binary variables. Individuals with the evidence of motion artifacts (mild and severe), less than ideal positioning (mildly asymmetric and rotated), or instability (mildly unstable and unstable) were placed in one grade whilst remaining individuals were included in the other.

Univariate logistic regression was used to screen potential covariates, including age, body weight, sex, dental disease, and auricular disease. Significance testing was performed using the Wald test. To account for repeated measures across imaging phases, final mixed‐effects models included interaction terms between restraint method and study phase. Those variables with a *p* value of less than 0.20 were selected for inclusion in a multivariate model. Model selection was performed using Akaike's Information Criterion (AIC) and backward elimination, with variables retained if their removal resulted in a >20% change in effect estimates.

Model diagnostics included assessment of residuals using simulation‐based diagnostics (DHARMa), evaluation of overdispersion, collinearity assessment using variance inflation factors, and influence diagnostics using Cook's distance and DFBETAs.

Odds ratios and 95% confidence intervals for main effects and interaction terms were derived from the fitted model on the log‐odds scale and estimated using estimated marginal means with pairwise contrasts. Confidence intervals were obtained using the delta method on the logit scale and back‐transformed to the odds ratio scale.

Model‐based predicted probabilities from the three logistic regression models were extracted and plotted to compare expected outcome probabilities across restraint methods.

## 3 Results

### 3.1 Study Population

Ninety‐seven rabbit CT studies fulfilled the inclusion criteria. The skull was included in all available studies. Four were excluded as follows: three repeat studies and one with incomplete data. Of the 93 studies, 36 were restrained in bunny burritos, 47 within the plexiglass chamber, and 10 with sedation. There was a transition from plexiglass chamber method to bunny burrito method at the end of 2022. The temporal shift of restraint methods and number of CT examinations over time is illustrated in Supporting Information S2.

Of all the bunny burrito studies included, 23/36 were from Institution 1, 10/36 were from Institution 2, and 3/36 were from Institution 3. Two larger sized rabbits were wrapped in bunny burrito over a foam wedge as they were too large for the plastic box, with similar image quality outcomes as the other burrito‐restrained rabbits. All sedation studies were from Institution 3: 8/10 received alpha‐2 agonists in combination with other agents, for complete list see Supporting Information S1.

Within each restrain method group, 24, 30, and 10 studies had intravenous contrast included, respectively. Most of these studies had two post‐contrast acquisitions. Additional acquisitions were made in two rabbits restrained in bunny burrito and seven rabbits in plexiglass chamber due to nondiagnostic images, and five rabbits in sedation group due to dacryocystography. The medians and quartile (Q1–Q3) of study duration of the studies with IV contrast were 8.0 (7.01–10.8), 8.0 (6.7–13.2), and 23.1 (15.6–26.9) min for bunny burrito, plexiglass chamber, and sedation, respectively. For studies without IV contrast, the medians and interquartile ranges were 16.5 (14.0–18.8) and 16.0 (14.0–17.0) s for bunny burrito and plexiglass chamber groups, respectively. The longer duration of contrast studies reflects their multiphase acquisition, but also time required for potential catheter placement, contrast administration and any additional procedures.

Demographic data of the rabbits are summarized in Table 1. The age (median: 57 months, Q1–Q3: 36–80 months) and body weight (median: 1.9 kg, Q1–Q3: 1.6–2.1 kg) of individuals were considered similar, with minimal variation in number of individuals across the different sexes and breeds between the restraint methods. Lop eared (*n* = 52) and mixed‐breed (*n* = 17) rabbits were the majority. Other breeds included Rex (*n* = 6), Lionhead (*n* = 6), Dutch (*n* = 2), Netherland Dwarf (*n* = 2), Flemish Giant (*n* = 2), Angora (*n* = 1), and Checkered Giant (*n* = 1), with less than three rabbits of a same breed in each restraint group.

**TABLE 1:** Subject demographics.

Variable	Restraint method			*p* value
	Bunny burrito	Plexiglass chamber	Sedation	
**Number of studies *n* (*n%*) *n* = 93**	36 (39)	47 (50)	10 (11)	
**Age** (months) *n* = 90	64	55	59	0.78
Median (Q1‐Q3)	(37‐84)	(36‐77)	(46‐77)	
**Bodyweight** (kg) *n* = 81	1.92	1.98	1.80	0.69
Median (Q1–Q3)	(1.70–2.10)	(1.62–2.21)	(1.67–2.06)	
**Gender** *n* (*n*%)				0.88
Intact female *n* = 15	4 (27)	10 (67)	1 (6)	
Neutered female *n* = 26	11 (42)	12 (46)	3 (12)	
Intact male *n* = 15	8 (50)	6 (38)	2 (12)	
Neutered male *n* = 36	13	19 (53)	4 (11)	
**Breed** *n* (*n*%)				0.07
Lop eared *n* = 53	20 (38)	31 (58)	2 (4)	
Mixed breed *n* = 16	6 (37)	7 (44)	3 (19)	
Other *n* = 21	8 (38)	8 (38)	5 (24)	

*Note: p* values are calculated using Fisher's exact test. Missing data were observed for age (*n* = 3), body weight (*n* = 12), gender (*n* = 1), and breed (*n* = 3). Missingness in bodyweight differed by restraint type (*p* = 0.03), with higher proportion missing in burrito‐restrained rabbits (25%) compared with plexiglass chamber (6%).

### 3.2 CT Image Quality and Findings

All 93 included studies were diagnostic according to the imaging reports. As mentioned above, a small subset of studies required additional acquisitions to achieve diagnostic standard. Nondiagnostic acquisitions were likely not systematically sent to the PACS. No complication was documented during or 24 h after CT examination.

The presenting complaints and the main CT diagnoses are described in Table 2. Supporting Information S3 summarizes the associations between presenting complaints and imaging diagnosis. Both dental disease and auricular disease were more frequently identified at CT diagnosis (over half of the patients) than would be expected on the basis of presenting complaints.

**TABLE 2:** Presenting complaints and CT‐diagnosed disease.

	Restraint method			*p* value
	Bunny burrito	Plexiglass chamber	Sedation	
**Presenting complaint type**				
**Auricular** *n* (*n*%)				0.29
Yes *n* = 41	16 (39)	21 (51)	4 (10)	
No *n* = 52	20 (38)	26 (50)	6 (12)	
**Dental** *n* (*n*%)				0.29
Yes *n*= 9	4 (33)	3 (56)	2 (11)	
No *n* = 84	32 (38)	44 (52)	8 (10)	
**Gastrointestinal** *n* (*n*%)				0.18
Yes *n* = 10	2 (40)	8 (60)	0 (0)	
No *n* = 83	34 (41)	39 (47)	10 (12)	
**Ocular** *n* (*n*%)				0.08
Yes *n* = 17	3 (18)	11 (64)	3 (18)	
No *n* = 76	33 (44)	36 (47)	7 (9)	
**Respiratory** *n* (*n*%)				0.38
Yes *n* = 20	7 (35)	9 (45)	4 (20)	
No *n* = 73	29 (40)	38 (52)	6 (8)	
**Other** *n* (*n*%)				0.78
Yes *n* = 9	4 (4)	5 (56)	0 (0)	
No *n* = 84	32 (38)	42 (50)	10 (12)	
**CT‐diagnosed disease type**				
**Dental disease** *n* (*n*%)				0.00037
Yes *n* = 53	14 (26)^α^	36 (68)^αβ^	3 (6)^β^	
No *n* = 40	22 (55)^α^	11 (28)^αβ^	7 (18)^β^	
**Auricular disease** *n* (*n*%)				0.79
Yes *n* = 52	20 (38)	27 (52)	5 (10)	
No *n* = 42	17 (40)	20 (48)	5 (12)	

*Note*: *p* values were calculated using Fisher's exact test. Tests for the presenting complaints were adjusted for multiple comparisons using the Holm method. Superscript letters indicate results of adjusted post‐hoc pairwise comparisons for disease type. α indicates significant difference between bunny burrito and plexiglass chamber (*p* = 0.002); β indicates significant difference between plexiglass chamber and sedation (*p* = 0.015). Two patients had no reported presenting complaint. As individual patients could present with multiple complaints, each reported symptom was included and analyzed as a separate observation.

There was a proportionally higher number of individuals with CT‐diagnosed dental disease in the plexiglass chamber group. Although this imbalance resulted in modest confounding, adjustment for dental disease consistently had only a limited impact on the estimated effects of restraint method. For good positioning, the estimated odds ratio for the bunny burrito method decreased from 3.22 to 2.91 after adjustment for dental disease. The odds of good stability and no motion artifacts were increased from 2.94 to 3.72 and 3.73 to 4.09. A significant association was observed between restraint method and dental disease status on CT. The proportion of rabbits with auricular disease was similar across bunny burrito, plexiglass chamber, and sedation groups. Abnormal soft tissue or mineral attenuations were identified in the nasal cavity of eight rabbits. Four rabbits had imaging findings compatible with dacryocystitis.

**FIGURE 4:**
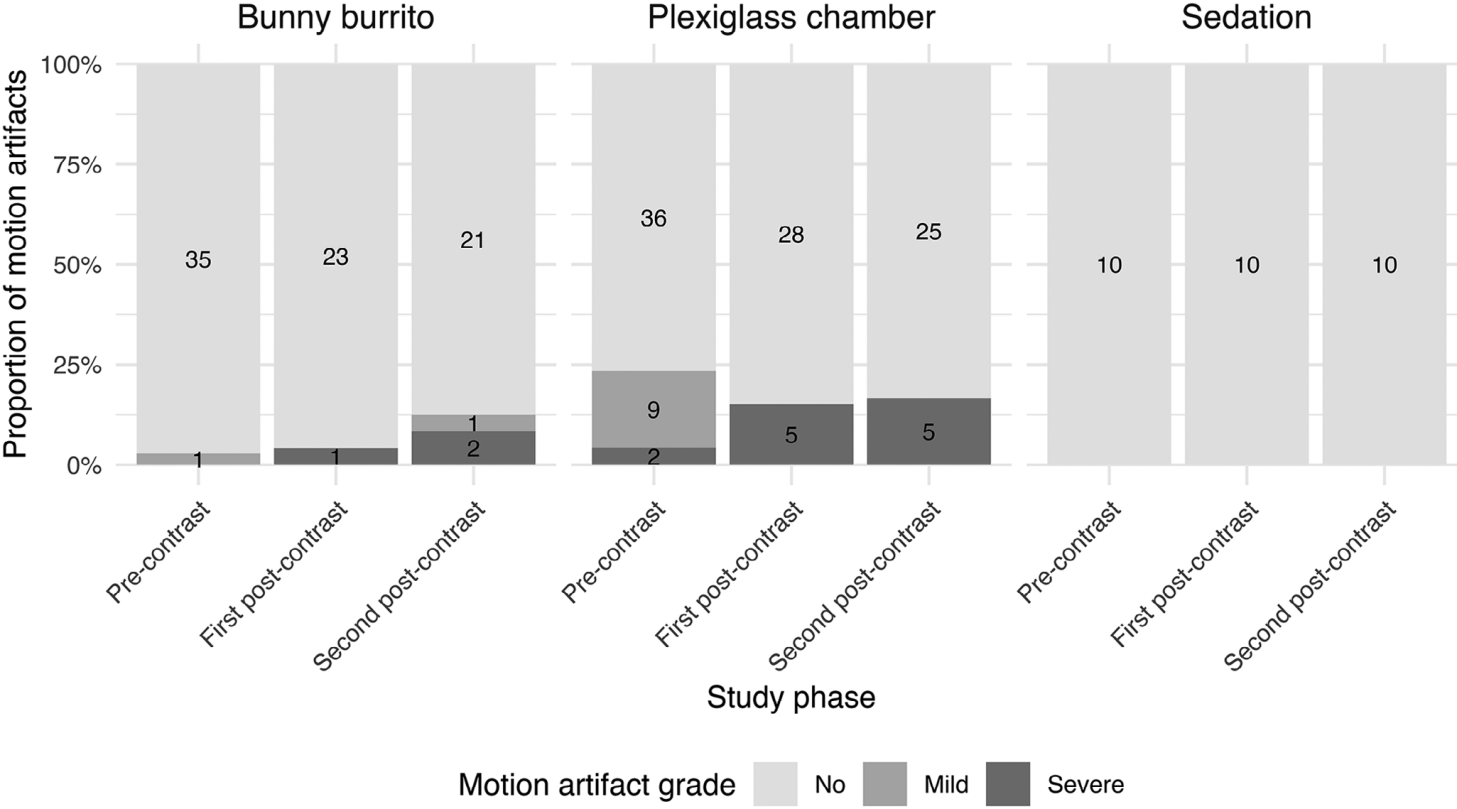
Proportional grades of motion artifact by restraint method and study phase.

**FIGURE 5:**
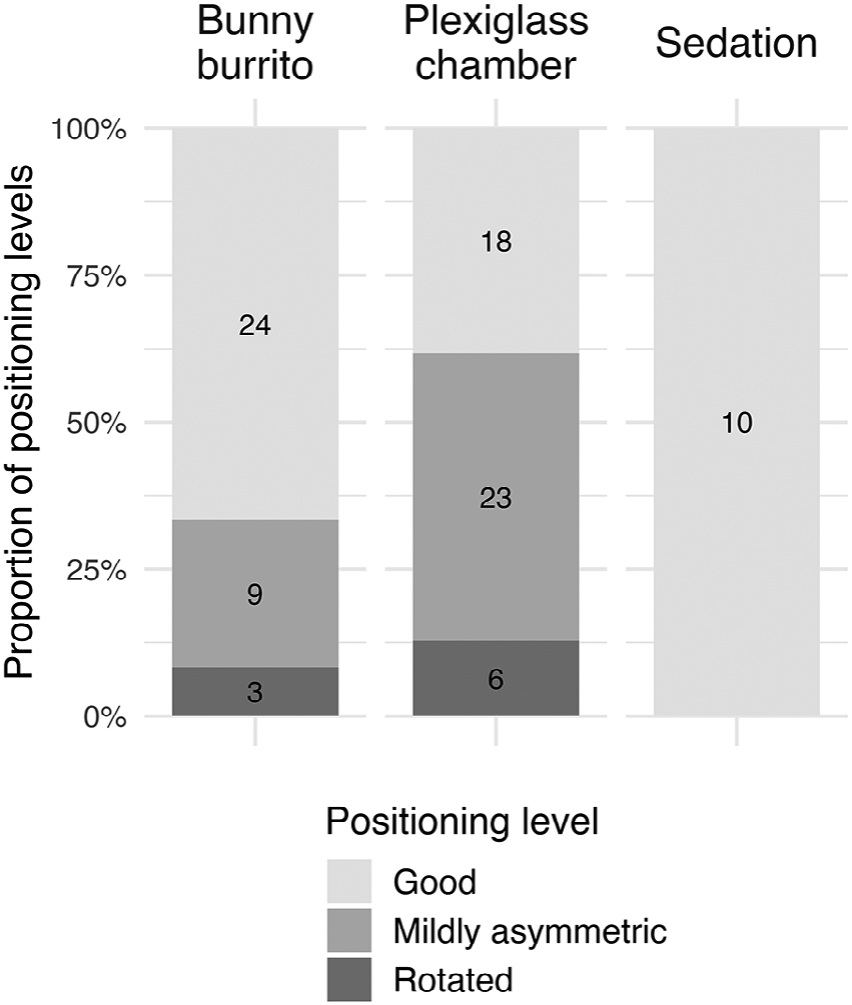
Proportional levels of positioning by restraint method.

**FIGURE 6:**
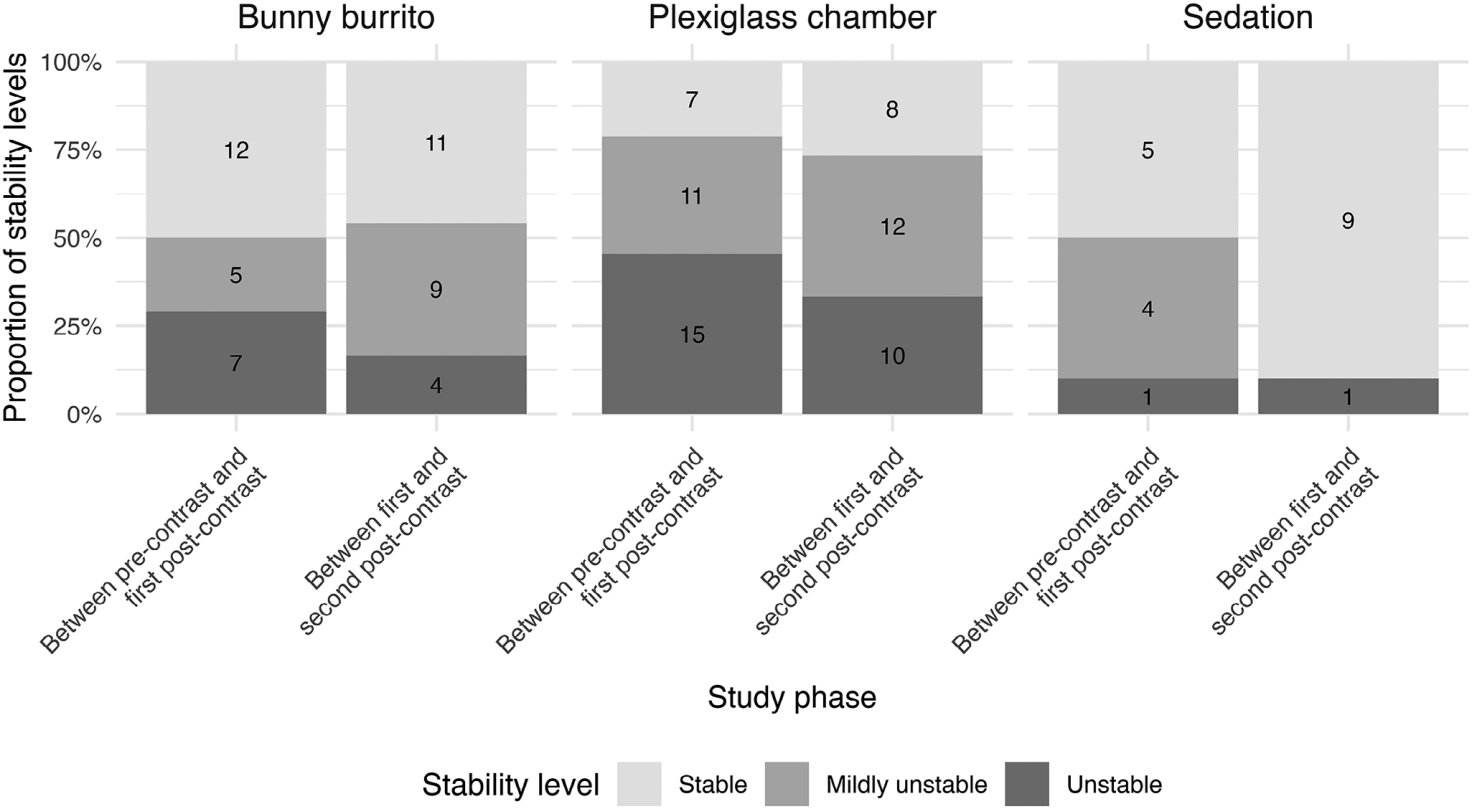
Proportional levels of stability by restraint method and study phase.

The bunny burrito method resulted in minimal motion artifacts, with fewer artifacts than the plexiglass chamber method, particularly in pre‐contrast and first post‐contrast acquisitions. The sedation method resulted in no motion artifacts (Figure 4). Thirty‐two (89%) of bunny burrito studies had no motion artifacts for the entire study duration, compared to 31 (66%) for the plexiglass chamber. The bunny burrito method had a higher proportion of good positioning (67%) than the plexiglass chamber (38%). The sedation method had consistently good positioning (Figure 5). The bunny burrito method led to a higher proportion of stable acquisitions than the plexiglass chamber method both between pre‐contrast and first post‐contrast, and first and second post‐contrast phases. The bunny burrito studies were stable for the entire duration of the study in eight (33%) studies and inconsistently stable in a further seven (29%) studies compared to four (12%) and seven (21%) for the plexiglass chamber. The sedation method resulted in 5/10 stable acquisitions between pre‐contrast and first post‐contrast and 9/10 stable acquisitions between first and second post‐contrast (Figure 6).

### 3.3 Comparison Between Bunny Burrito and Plexiglass Chamber

Table 3 presents regression results comparing bunny burrito and plexiglass chamber restraint methods across study phases. The interaction term was retained in the mixed‐effect models due to a ∼20% change in the estimated effect of restraint when excluded.

**TABLE 3:** Comparison of the bunny burrito and plexiglass chamber restraint methods using multivariable logistic regression.

Parameter of study quality	Subgroup	Odds ratio	95% CI	*p* value
**Motion artifacts** ^a^				
Pre‐contrast *n* = 83		0.06	0.01–0.69	0.02*
First post‐contrast *n* = 57		0.19	0.01–2.63	0.21
Second post‐contrast *n* = 54		0.63	0.09–4.66	0.65
**Positioning** ^b^ *n* = 80				
	Without dental disease	0.80	0.31–2.04	0.64
	With dental disease	12.30	3.39–44.26	0.03*
**Stability** ^a^				
Between pre‐contrast and first post‐contrast *n* =57		8.33	1.09–63.90	0.04*
Between first post‐contrast and second post‐contrast *n* = 54		4.26	0.61–29.60	0.14

*Note*: Outcomes included were motion artifacts, positioning quality, and inter‐acquisition stability. Odds ratios represent bunny burrito relative to plexiglass chamber (reference).

^a^CT study phase‐specific odds ratios were derived from models including an interaction between phase and restraint, with rabbit ID included as a random intercept.

^b^Adjusted for age.

^*^Statistically significant. Missing data were observed for age (*n* = 3) in the positioning group. Rabbits without first post‐contrast (*n* = 26) and second post‐contrast (*n* = 28) imaging were excluded from the respective motion artifact and stability analyses.

Overall, the bunny burrito restraint was associated with lower odds of motion artifacts in pre‐contrast images compared to the plexiglass chamber. However, effects in later phases were attenuated and no longer significantly different.

For positioning, increasing age was associated with slightly lower odds of successful positioning (OR = 0.97, 95% CI: 0.96–0.99, *p* = 0.009). The bunny burrito method was associated with higher odds of correct positioning in rabbits with dental disease, whereas no significant difference was found for those without. Rabbits with dental disease may benefit more from the body alignment control and physical support afforded by the bunny burrito method.

Between pre‐ and first post‐contrast phases, bunny burrito restraint was associated with greater odds of good stability, with attenuation in later phases.

**FIGURE 7:**
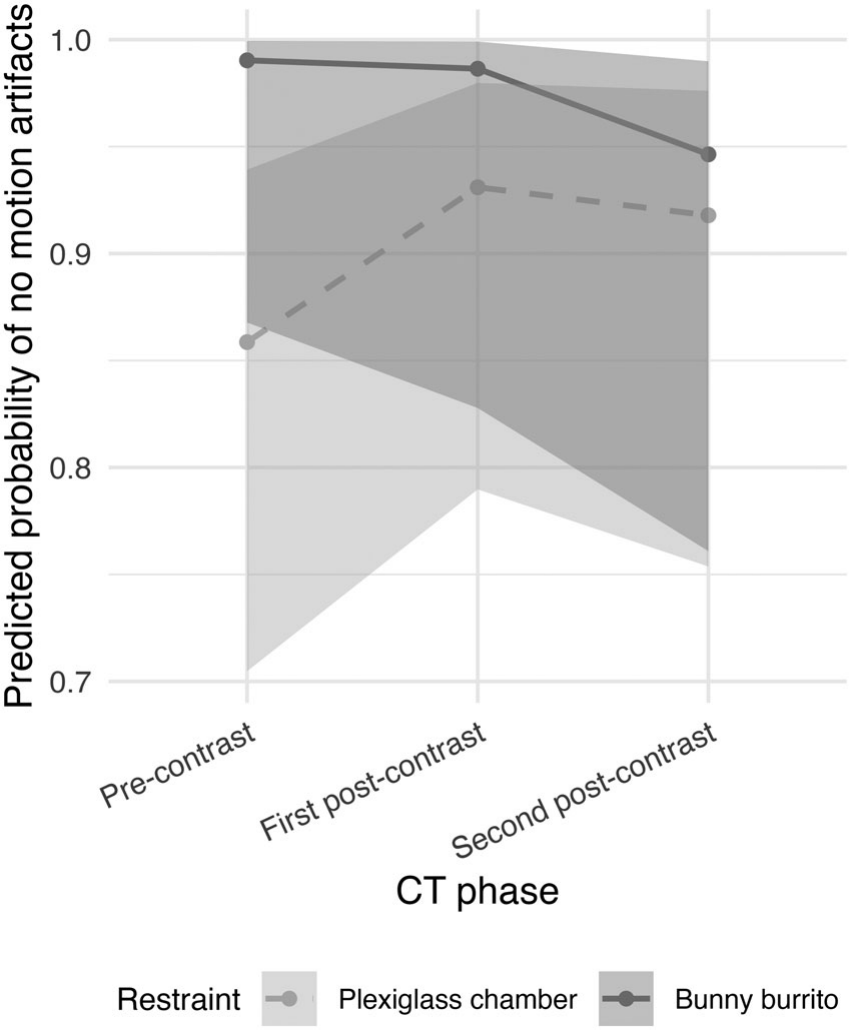
Predicted probability of no motion artifacts over time for plexiglass chamber and bunny burrito restraint methods. Sample sizes were *n* = 83 (pre‐contrast), *n* = 57 (first post‐contrast), and *n* = 54 (second post‐contrast). Lines represent model‐based predicted probabilities, with shaded ribbons indicating 95% confidence intervals.

**FIGURE 8:**
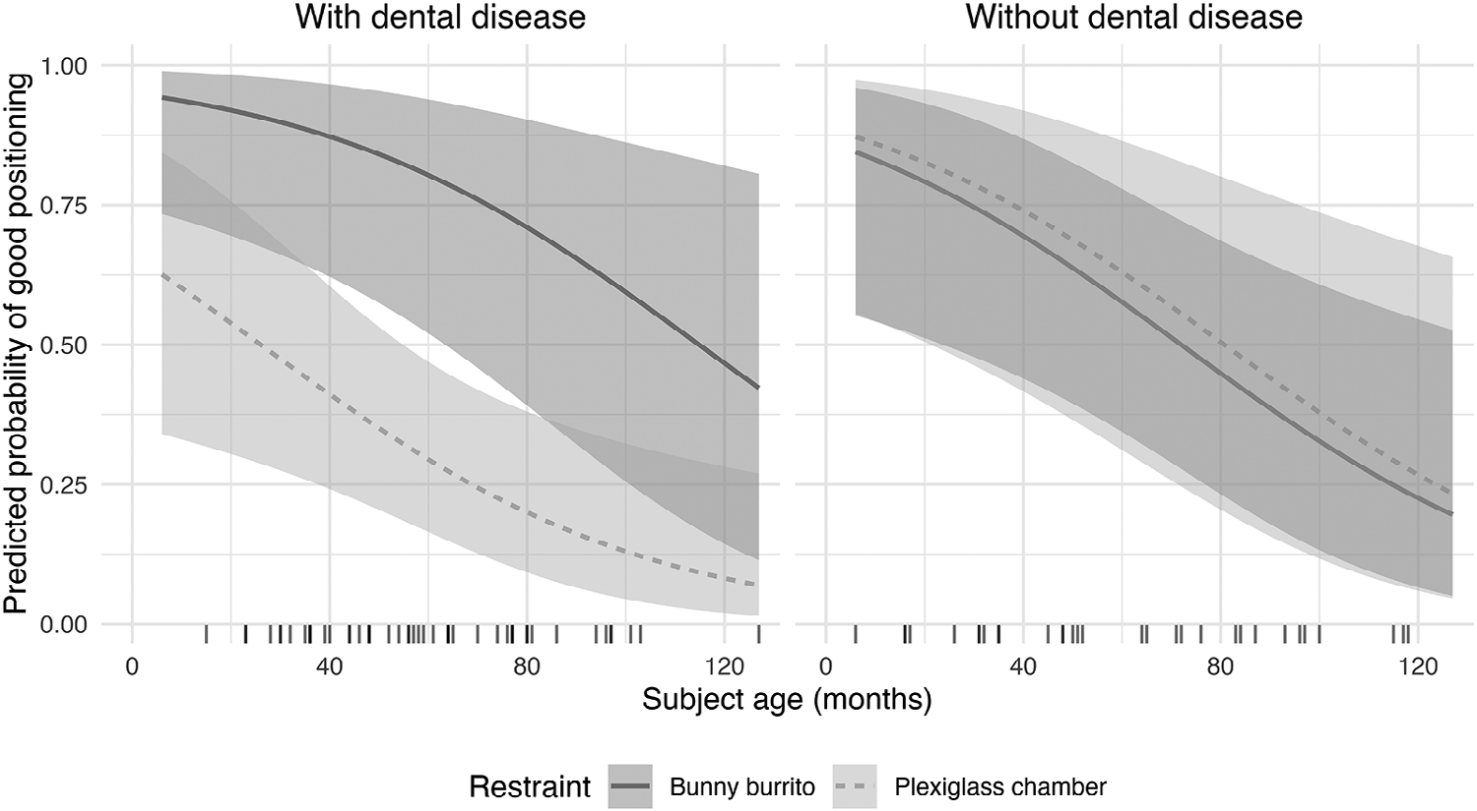
Predicted probability of good positioning in rabbits with or without dental disease over subject age for plexiglass chamber and bunny burrito restraint methods. Sample sizes were *n* = 50 (with dental disease) and *n* = 33 (without dental disease). Lines represent model‐based predicted probabilities, with shaded ribbons indicating 95% confidence intervals. Rug plots along the *x*‐axis indicate the distribution of ages within each group, with darker ticks reflecting overlapping observations.

**FIGURE 9:**
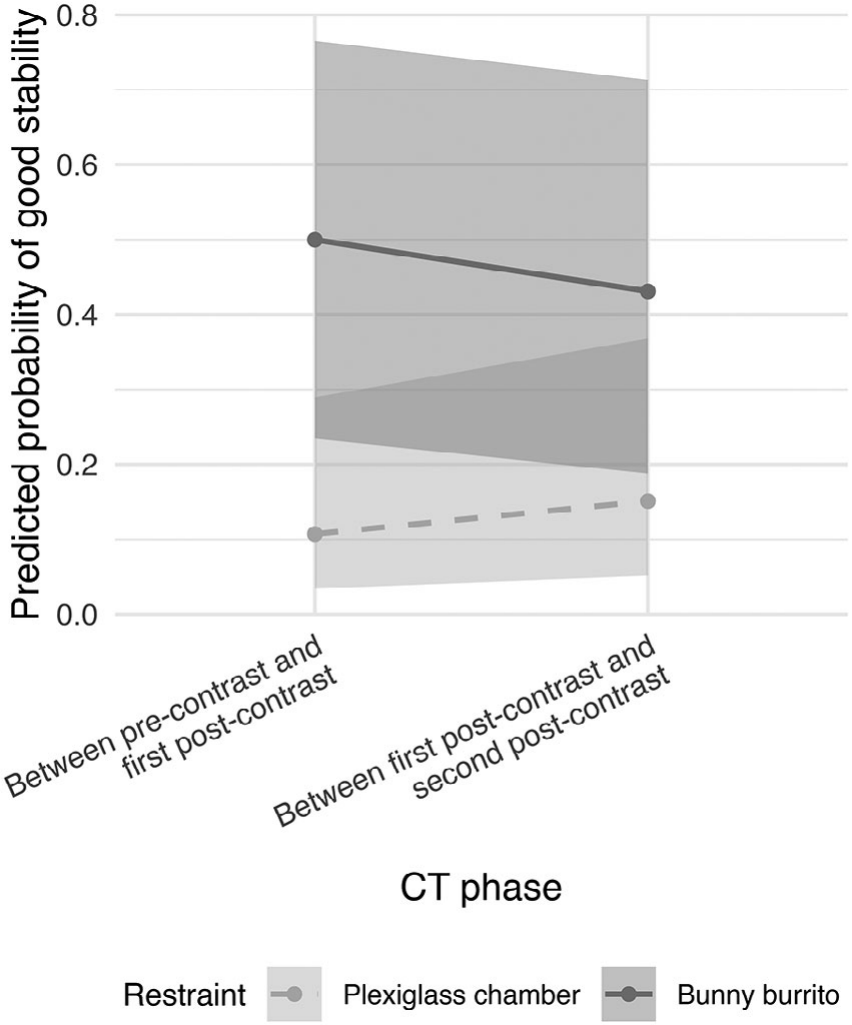
Predicted probability of good stability over time for plexiglass chamber and bunny burrito restraint methods. Sample sizes were *n* = 57 (pre‐to‐first post‐contrast comparison) and *n* = 54 (first‐to‐second post‐contrast comparison). Lines represent model‐based predicted probabilities, with shaded ribbons indicating 95% confidence intervals.

Figures 7, 8, 9 describe the predicted probabilities of no motion artifacts, good positioning, and good stability, respectively.

To address the possible confounding factors, additional analyses were conducted for differences in dental disease status, study date, institution, and high‐pitch acquisitions. The number of CTs performed increased by 63% per year (IRR: 1.63, 95% CI: 1.26–2.14, *p* = <0.001), while the proportion of rabbits diagnosed with dental disease changed over time. There was a slight increase in the proportion of rabbits CT‐diagnosed with dental disease prior to December 2022 (OR: 1.004, 95% CI: 1.001–1.008, *p* = 0.05) followed by a significant decrease thereafter (OR: 0.992, 95% CI: 0.985–0.998, *p* = 0.01).

Dental disease was found to be associated with positioning outcomes only (Table 3). No significant associations were observed for motion artifacts (*p* = 0.72) or stability (*p* = 0.27).

Adding a time‐centered annual variable as a fixed effect in the final regression models showed no statistically significant evidence of temporal bias in movement (*p *= 0.73), positioning (*p *= 0.91), or stability (*p* = 0.61) over the study period.

When adjusting for institution (Institutions 1, 2, and 3), no significant differences were observed for motion artifacts (Institution 2, *p* = 0.48; no motion artifacts recorded for Institution 3), positioning quality (Institution 2, *p* = 0.22; Institution 3, *p *= 0.61), or stability (Institution 2, *p* = 0.69; Institution 3, *p *= 0.23).

No significant difference between high and normal pitch was observed for any of the three outcome measures (motion artifact: *p* = 0.24; stability: *p* = 0.10; positioning: *p* = 0.87).

## 4 Discussions

This study constitutes the first description of a novel use of the well‐known bunny burrito technique in a new setting: head CT examination of conscious rabbits. Although this very popular technique is mostly described and recommended for physical examination and medication, this study has shown its use for physical restraining during CT acquisition. According to our literature review, this study is also the first to evaluate the effectiveness of restraint methods for conducting CT examinations in rabbits. Two physical restraint techniques were compared as follows: the bunny burrito and the plexiglass chamber methods, with additional descriptive comparisons made to sedation. The results partially support our hypothesis that the bunny burrito method outperformed the plexiglass chamber: specifically, by reducing motion artifacts in pre‐contrast phase, having superior positioning in rabbits with dental disease, and enhancing stability during pre‐ to early post‐contrast period. In other acquisition phases and disease context, the burrito method is similar to the plexiglass chamber method, which had already been proven as suitable for conscious CT imaging of rabbits [12, 13, 15]. No statistical comparison with sedation group was performed due to small sample size; based on a small descriptive comparison limited to one institution, the bunny burrito method achieved comparable motion‐free acquisitions with slightly higher oblique positioning and slightly lower stability. Across all included cases, imaging diagnoses were successfully achieved, suggesting that the bunny burrito method was an effective approach for CT studies of the skull in conscious rabbits.

Using physical restraint on conscious rabbits eliminates the risks associated with sedation and anesthesia, such as chemically induced mortality and gastrointestinal stasis [8, 9]. Despite efforts to minimize stress through dim lighting, low ambient noise, and contention in natural resting position, physical restraint during CT remains a source of stress. However, no adverse events were reported during or within 24 h post‐examination in the bunny burrito group, demonstrating its safety for conscious imaging. The other advantage of physical restraint is that, as there is no recovery phase, rabbits can be discharged directly after scanning, improving efficiency of the procedure and lowering costs. The bunny burrito method is also accessible and practical, requiring only a towel and an open‐top box. The towel ensures gentle restraint, whereas the box provides stability and comfortably anchors the mandible in a physiological position. Compared to the plexiglass chamber, this method is simpler to set up, without need for additional oxygen support as the rabbits have direct access to air, making it more feasible in varied clinical settings. Additionally, the open‐top box allows easier access for procedures, such as contrast injection, catheter adjustment, and dacryocystography. The high accessibility and low cost mean that this technique could extend the utility of CT to more rabbits, especially for common conditions like dental and auricular diseases. Manual contrast injection allowed personnel to maintain close monitoring of the patient as conscious rabbits may exhibit occasional movement during injections. While standing alongside the rabbits during injections, the operators can promptly correct any positional changes in between acquisitions.

Motion artifacts, positioning, and stability are important factors that affect the imaging interpretation process. Bunny burrito method achieved 89% of motion‐free and 67% of ideally positioned acquisitions. Symmetrical positioning helps in evaluating anatomical structures and pathology. Although multiplanar reconstruction allows subsequent orientation adjustments for accurate regions‐of‐interest comparison, these manual adjustments increase interpretation time. Workload in radiology and particularly CT is increasing, supporting the establishment of techniques decreasing acquisition and interpretation time while ensuring patient safety. The bunny burrito method allows easy access for the operator to adjust the position before the scans, as the rabbits are not enclosed in a container like in a plexiglass chamber. Dental disease on CT was found to be a significant predictor of correct positioning outcomes and was present in over half the cohort. As no association was identified between dental disease and movements, the interaction with positioning may instead reflect the greater handler control over body alignment afforded by tight wrapping. The full body wrapping may have a calming effect on rabbits with dental disease, possibly more stressed due to chronic pain. Although age was associated with a slight decrease in ideal positioning, the odds ratio was close to one, indicating a very mild effect. This may be influenced by age‐related comorbidities; further prospective research is needed to validate this hypothesis.

The stability of patient between acquisitions is important when comparing the contrast‐enhancing features of the region of interest, and it has not been evaluated in conscious rabbit CT before. Having the subject stay at the same location between the scans, the different studies can then directly be synchronized and compared in the viewing software. The towel wrap in bunny burrito method provides gentle but firm restraint to prevent rabbits from moving. In contrast, the towel used in the plexiglass chamber acted more as a soft cushion or space‐occupying material, which might lack stability. One rabbit in the plexiglass chamber group turned 180° facing the other end between the scans. A carefully wrapped rabbit may have been one of the critical factors contributing to the enhanced stability of bunny burrito method. The same feature likely contributed to the reduction of motion artifacts when using the bunny burrito method. The lower stability in sedation group cannot be generalized due to the small sample size and possible institutional bias. The changes detected between acquisitions in this group were, however, mostly mild, possibly due to intermittent monitoring.

This study has several limitations. First, awareness of the hypothesis during data extraction could have introduced extraction bias. Due to the visibility of retraining devices in the field of view, a blinded evaluation was not possible. Therefore, bias during grading could not be fully excluded. The retrospective design may have introduced bias, potentially underestimating motion artifacts and overestimating oblique positioning. Indeed, some acquisitions with severe artifacts may not have been uploaded to the PACS. Positioning defects may also have been overestimated in the group of rabbits (*n* = 10) acquired from one of the centers where the CT system allowed oblique reconstructions, so a symmetric position was not necessary for this group. Personnel heterogeneity may introduce variability as bunny burrito is technique‐dependent. However, burrito wrapping is a widespread and simple technique that all involved personnel in this study had been trained in. This study focused solely on skull imaging, benefits may differ for other anatomical regions, warranting further investigation.

In conclusion, the bunny burrito is a simple and effective restraint technique for CT examination of the skull in conscious rabbit. It showed similar or superior performance to the plexiglass chamber method results across all evaluated phases, with targeted advantages in motion reduction, positioning, and potential inter acquisition stability while similarly avoiding sedation‐related risks.

## Author Contributions

Conception and design: Hung‐Ting Liu, Laetitia Volait‐Rosset, and Alexis Gombert. Acquisition of data: Hung‐Ting Liu, Ellen Sundberg, and Alexis Gombert. Analysis and interpretation of data: Hung‐Ting Liu, Jonathan Olijnyk, Jessica Ingman, and Alexis Gombert. Drafting the article: Hung‐Ting Liu, Jonathan Olijnyk, and Alexis Gombert. Revising article for intellectual content: Hung‐Ting Liu, Jonathan Olijnyk, Jessica Ingman, Ellen Sundberg, Laetitia Volait‐Rosset, and Alexis Gombert. Final approval of the completed article: Hung‐Ting Liu, Jonathan Olijnyk, Jessica Ingman, Ellen Sundberg, Laetitia Volait‐Rosset, and Alexis Gombert.

## Disclosure

An abstract has been presented as oral presentation for EVDI congress September 19th to 21st 2024 in Greece. The STROBE checklist was followed where applicable.

## Conflicts of Interest

The authors declare no conflicts of interest.

## Supporting information




**Supporting Information S1**: Sedation protocols.
**Supporting Information S2**: Predicted probabilities of restraint methods and actual number of CT examinations over the study period.
**Supporting Information S3**: Associations between presenting clinical signs and final diagnosis for dental and auricular disease.
**Supporting Information S4**: Univariate logistic regression analysis results for motion artifacts.
**Supporting Information S5**: Univariate logistic regression analysis results for positioning.
**Supporting Information S6**: Univariate logistic regression analysis results for stability.
**Supporting Information S7**: Mixed‐effects logistic regression analysis results for motion artifacts.
**Supporting Information S8**: Multivariate logistic regression analysis results for positioning.
**Supporting Information S9**: Mixed‐effects logistic regression analysis results for stability.
**Supporting Information S10**: Final regression models.

## Data Availability

The data are available from the corresponding author upon reasonable request.
