# Supplementary material for: Bunny Burrito Restraint for Computed Tomography in Conscious Rabbits: An Effective Alternative
Source: Vet Radiol Ultrasound. 2026 Aug 26;67(5):e70230. doi: 10.1111/vru.70230 (PMC13510107; doi:10.1111/vru.70230)
Supplement: Supplementary file 1 [file VRU-67-0-s001.docx]

Bunny burrito restraint for computed tomography in conscious rabbits: An effective alternative

Supporting information

S1: Table 1: Sedation protocols

| Rabbit identification in sedation group | Study date  (year-month-day) | Sedation protocol (intramuscular injection) |
| --- | --- | --- |
| 1 | 2021-12-08 | Medetomidine (0.1 mg/kg), midazolam (1 mg/kg), alfaxalone (4 mg/kg). |
| 2 | 2023-02-22 | Dexmedetomidine (0.06 mg/kg), midazolam (1 mg/kg), butorphanol (0.3 mg/kg) |
| 3 | 2023-04-11 | Dexmeditomidine (0.06 mg/kg), midazolam (1 mg/kg) |
| 4 | 2023-05-10 | Medetomidine (0.1 mg/kg), midazolam (1 mg(kg), butorphanol (0.3 mg/kg) |
| 5 | 2023-05-10 | Butorphanol (0.3 mg/kg), midazolam (1 mg/kg) |
| 6 | 2023-05-10 | Medetomidine (0.1 mg/kg), midazolam (1 mg/kg), butorphanol (0.3 mg/kg) |
| 7 | 2023-06-16 | Dexmeditomidine (0.06 mg/kg), midazolam (1 mg/kg), butorphanol (0.3 mg/kg) |
| 8 | 2023-06-28 | Medetomidine (0.1 mg/kg), midazolam (1 mg/kg), butorphanol (0.3 mg/kg) |
| 9 | 2023-06-28 | Medetomidine (0.1 mg/kg), midazolam (1 mg/kg), butorphanol (0.3 mg/kg) |
| 10 | 2023-09-14 | Midazolam (1 mg/kg) |

Intramuscular sedation agents:

- Medetomidine (Sedator vet; Dechra)
- Dexmedetomidine (Dexdomitor; Zoetis)
- Butorphanol (Dolorex; MSD Animal Health or Torbudine; Dechra)
- Midazolam (Dormaquin; Dechra)

Intramuscular sedation reversion agents:

- Atipamezole (Atipam; Dechra) 0.5 mg/kg
- flumazenile (Flumazenil; Hameln) 1 mg/kg

S2: Figure 1: Predicted probabilities of each restraint method as a function of time, estimated from a multinominal logistic regression model with centered time. Lines show the predicted probability of each method, with shaded grey bands representing 95% confidence intervals. The histogram below shows the density of CT examinations over the study period.

S3: Table 2: Associations between presenting clinical signs and final diagnosis for dental and auricular disease

|  | **Diagnosed Disease** | | | | | |
| --- | --- | --- | --- | --- | --- | --- |
|  | **Auricular Disease** | | | **Dental Disease** | | |
| **Presenting sign category** | **OR** | **95% CI** | **p-value** | **OR** | **95% CI** | **p-value** |
| Auricular |  |  |  | 1.26 | 0.39 – 4.15 | 0.695 |
| Dental | 0.09 | 0.004 – 0.59 | 0.033* |  |  |  |
| Gastrointestinal | 0.38 | 0.08 – 1.58 | 0.186 | 2.48 | 0.65 – 10.99 | 0.199 |
|  |  |  |  |  |  |  |
| Neurological | 12.42 | 2.06 – 240 | 0.022* | 0.65 | 0.21 – 1.99 | 0.451 |
| Ocular | 0.38 | 0.09 – 1.43 | 0.164 | 1.63 | 0.42 – 6.60 | 0.484 |
| Respiratory | 0.14 | 0.03 – 0.49 | 0.004* | 0.56 | 0.15 – 1.99 | 0.365 |

Odds ratios (OR) and 95% confidence intervals (95% CI) were derived from multivariable logistic regression models, with presenting signs entered as binary predictors. p-values were calculated using Wald z-tests.

| **Variable** | **Comparison** | **Odds Ratio (95%CI)** | **P-value** |
| --- | --- | --- | --- |
| **Auricular Dis.** |  |  |  |
| Yes | No | 0.500 (0.100 - 1.943) | 0.344 |
| **Age** |  | 0.991 (0.969 - 1.014) | 0.456 |
| **Body Weight** |  | 1.166 (0.386 - 5.322) | 0.808 |
| **Dental Dis.** |  |  |  |
| Yes | No | 0.750 (0.178 - 2.765) | 0.674 |
| **Restraint Method** |  |  |  |
| Burrito | Plexiglass Chamber | 11.500 (1.986 - 219.150) | 0.025 |
| Plexiglass Chamber | Burrito | 0.087 (0.005 - 0.503) | 0.025 |
| **Sex** |  |  |  |
| Entire Female | Entire Male | 2.306e-08 (NA - 4.837e+61) | 0.994 |
| Entire Female | Neutered Female | 0.889 (0.153 - 5.544) | 0.895 |
| Entire Female | Neutered Male | 0.833 (0.160 - 4.891) | 0.830 |
| Entire Male | Entire Female | 4.334e+07 (2.067e-62 - NA) | 0.994 |
| Entire Male | Neutered Female | 3.855e+07 (2.652e-102 - NA) | 0.994 |
| Entire Male | Neutered Male | 3.614e+07 (1.722e-62 - NA) | 0.994 |
| Neutered Female | Entire Female | 1.125 (0.180 - 6.521) | 0.895 |
| Neutered Female | Entire Male | 2.594e-08 (NA - 4.367e+70) | 0.994 |
| Neutered Female | Neutered Male | 0.938 (0.204 - 4.505) | 0.933 |
| Neutered Male | Entire Female | 1.200 (0.204 - 6.260) | 0.830 |
| Neutered Male | Entire Male | 2.767e-08 (NA - 1.262e+52) | 0.994 |
| Neutered Male | Neutered Female | 1.067 (0.222 - 4.900) | 0.933 |
| **Study Duration** (minutes) |  | 0.970 (0.898 - 1.035) | 0.320 |

S4: Table 3: Univariate Logistic Regression Analysis Results for Motion Artifacts

S5: Table 4: Univariate Logistic Regression Analysis Results for Positioning

| **Variable** | **Comparison** | **Odds Ratio (95%CI)** | **P-value** |
| --- | --- | --- | --- |
| **Auricular Disease** |  |  |  |
| Yes | No | 0.857 (0.357 - 2.046) | 0.729 |
| **Age** |  | 0.978 (0.960 - 0.995) | 0.013 |
| **Body Weight** |  | 1.170 (0.657 - 2.262) | 0.597 |
| **Dental Disease** |  |  |  |
| Yes | No | 0.511 (0.205 - 1.24) | 0.141 |
| **Restraint Method** |  |  |  |
| Burrito | Plexiglass Chamber | 3.222 (1.321 - 8.205) | 0.012 |
| Plexiglass Chamber | Burrito | 0.310 (0.122 - 0.757) | 0.012 |
| **Sex** |  |  |  |
| Entire Female | Entire Male | 1.167 (0.255 - 5.434) | 0.842 |
| Entire Female | Neutered Female | 0.769 (0.199 - 2.945) | 0.700 |
| Entire Female | Neutered Male | 1.133 (0.318 - 4.051) | 0.845 |
| Entire Male | Entire Female | 0.857 (0.184 - 3.928) | 0.842 |
| Entire Male | Neutered Female | 0.659 (0.163 - 2.590) | 0.550 |
| Entire Male | Neutered Male | 0.971 (0.259 - 3.567) | 0.965 |
| Neutered Female | Entire Female | 1.300 (0.340 - 5.031) | 0.700 |
| Neutered Female | Entire Male | 1.517 (0.386 - 6.144) | 0.550 |
| Neutered Female | Neutered Male | 1.473 (0.503 - 4.408) | 0.481 |
| Neutered Male | Entire Female | 0.882 (0.247 - 3.144) | 0.845 |
| Neutered Male | Entire Male | 1.029 (0.280 - 3.856) | 0.965 |
| Neutered Male | Neutered Female | 0.679 (0.227 - 1.987) | 0.481 |

S6: Table 5: Univariate Logistic Regression Analysis Results for Stability

| **Variable** | **Comparison** | **Odds Ratio (95%CI)** | **P-value** |
| --- | --- | --- | --- |
| **Auricular Disease** |  |  |  |
| Yes | No | 0.367 (0.114 - 1.139) | 0.085 |
| **Age** |  | 1.007 (0.987 - 1.028) | 0.473 |
| **Body Weight** |  | 0.472 (0.096 - 1.480) | 0.274 |
| **Dental Disease** |  |  |  |
| Yes | No | 1.118 (0.362 - 3.604) | 0.848 |
| **Restraint Method** |  |  |  |
| Burrito | Plexiglass Chamber | 3.714 (1.198 - 12.359) | 0.026 |
| Plexiglass Chamber | Burrito | 0.269 (0.081 - 0.835) | 0.026 |
| **Sex** |  |  |  |
| Entire Female | Entire Male | 0.457 (0.070 - 2.715) | 0.394 |
| Entire Female | Neutered Female | 1.714 (0.315 - 9.548) | 0.527 |
| Entire Female | Neutered Male | 1.429 (0.287 - 6.790) | 0.652 |
| Entire Male | Entire Female | 2.188 (0.368 - 14.349) | 0.394 |
| Entire Male | Neutered Female | 3.750 (0.683 - 23.39) | 0.135 |
| Entire Male | Neutered Male | 3.125 (0.626 - 16.991) | 0.168 |
| Neutered Female | Entire Female | 0.583 (0.105 - 3.179) | 0.527 |
| Neutered Female | Entire Male | 0.267 (0.043 - 1.464) | 0.135 |
| Neutered Female | Neutered Male | 0.833 (0.178 - 3.611) | 0.809 |
| Neutered Male | Entire Female | 0.700 (0.147 - 3.478) | 0.652 |
| Neutered Male | Entire Male | 0.320 (0.059 - 1.600) | 0.168 |
| Neutered Male | Neutered Female | 1.200 (0.277 - 5.624) | 0.809 |
| **Study Duration** (minutes) |  | 0.742 (0.567 - 0.917) | 0.014 |

S7: Table 6: Mixed-Effects Logistic Regression Analysis Results for Motion Artifacts

| **Variable** | **Effect**  **(Reference Condition)** | **Odds Ratio (95%CI)** | **P-value** |
| --- | --- | --- | --- |
| **Intercept** | Baseline  (Chamber, Pre-Contrast) | 0.16 (0.05 – 0.56) | 0.004 |
| **Restraint** |  |  |  |
| (Burrito) | Burrito vs Plexiglass Chamber  (Pre-Contrast) | 0.06 (0.01 – 0.69) | 0.024 |
| **Phase** |  |  |  |
| First Post-Contrast | First Post-Contrast  vs Pre-Contrast  (Plexiglass Chamber Only) | 0.45 (0.11 – 1.85) | 0.268 |
| Second Post-Contrast | Second Post-Contrast  vs Pre-Contrast  (Plexiglass Chamber Only) | 0.54 (0.13-2.25) | 0.400 |
| **Restraint x Phase** |  |  |  |
| First Post-Contrast | Modification of Restraint Effect at First Post-Contrast | 3.13 (0.12 – 84.22) | 0.497 |
| Second Post-Contrast | Modification of Restraint Effect at Second Post-Contrast | 10.67 (0.59 – 193.53) | 0.109 |

Table 6a – Mixed Effects Analysis for Motion Artifacts – fixed effects.

| **Random Effect** | **SD (95% CI)** |
| --- | --- |
| Rabbit ID (intercept) | 1.65 (0.74 – 3.67) |

Table 6b – Mixed Effects Analysis for Motion Artifacts – random effect.

S8: Table 7: Multivariate Logistic Regression Analysis Results for Positioning

| **Variable** | **Effect**  **(Reference Condition)** | **Odds Ratio (95%CI)** | **P-value** |
| --- | --- | --- | --- |
| **Age (centered)** | Per month increase | 0.97 (0.96 – 0.99) | 0.009 |
| **Dental Disease** |  |  |  |
| (Yes) | Yes vs No  (Plexiglass Chamber) | 0.25 (0.05 -1.08) | 0.071 |
| **Restraint Method** |  |  |  |
| (Burrito) | Burrito vs Plexiglass Chamber  (No Dental Disease) | 0.80 (0.15 – 4.07) | 0.787 |
| **Dental Disease and Restraint Method** | | | |
| Restraint x Dental disease | Modification of  restraint effect  (when dental disease = Yes) | 12.30 (3.39 – 44.26) | 0.028 |

S9: Table 8: Mixed-Effects Logistic Regression Analysis Results for Stability

| **Variable** | **Effect**  **(Reference Condition)** | **Odds Ratio (95%CI)** | **P-value** |
| --- | --- | --- | --- |
| **Intercept** | Baseline  (Chamber, Pre-Contrast to First Post-Contrast) | 8.34 (1.70 -40.86) | 0.009 |
| **Restraint** |  |  |  |
| Burrito | Burrito vs Plexiglass Chamber  (Pre-Contrast to First Post-Contrast) | 0.12 (0.02 – 0.92) | 0.041 |
| **Phase** |  |  |  |
| First to Second Post-Contrast | Pre-to-First vs First-to-Second Post Contrast  (Plexiglass Chamber Only) | 0.67 (0.16 – 2.81) | 0.588 |
| **Restraint x Phase** |  |  |  |
| First to Second Post-Contrast | Modification of Restraint Effect between First and Second Post-Contrast | 1.96 (0.25 – 15.23) | 0.521 |

Table 8a – Mixed Effects Analysis for Stability – fixed effects.

| **Random Effect** | **SD (95% CI)** |
| --- | --- |
| Rabbit ID (intercept) | 1.83 (0.83 – 4.09) |

Table 8b – Mixed Effects Analysis for Stability – random effect.

S10: Final Regression Models

$$logit(P(Positioning)=0.533-0.224\cdot Restraint-0.026\cdot\left( Age-59.963 \right)-1.404\cdot DD$$

$$+2.506\cdot Burrito\cdot DD$$

$$logit(P\left( Stability \right)=2.121-2.120\cdot Restraint-0.400\cdot FtoS+0.673\cdot\left( Restraint\cdot FtoS \right)+random\_intercept (Rabbit\_id)$$

$$logit\left( P\left( Motion Artifacts \right) \right)=-1.833-2.813\cdot Restraint-0.798\cdot Post1-0.616\cdot Post2+1.141\cdot\left( Restraint\cdot Post1 \right)+2.367\cdot\left( Restraint\cdot Post2 \right)+random\_intercept (Rabbit\_id)$$

$Restraint$ was coded as 0 for the plexiglass chamber and 1 for the bunny burrito. $Age$ represents the subject’s age in months. Dental disease ($DD$) was coded as 0 for absence and 1 for presence of dental disease. $PtoF$ is coded as 1 for the interval between the pre-contrast and first post-contrast phases and 0 otherwise. $FtoS$ is coded as 1 for the interval between the first and second post-contrast phases and 0 otherwise. $Post1$ is an indicator variable coded as 1 for the first post-contrast phase and 0 otherwise. $Post2$ is coded as 1 for the second post-contrast phase and 0 otherwise. $Rabbit\_id$ identifies individual rabbits and was included as a random effect in mixed-effects models.
